# Experimental and Theoretical Reduction Potentials of Some Biologically Active *ortho*-Carbonyl *para*-Quinones

**DOI:** 10.3390/molecules22040577

**Published:** 2017-04-04

**Authors:** Maximiliano Martínez-Cifuentes, Ricardo Salazar, Oney Ramírez-Rodríguez, Boris Weiss-López, Ramiro Araya-Maturana

**Affiliations:** 1 Programa Institucional de Fomento a la Investigación, Desarrollo e Innovación, Universidad Tecnológica Metropolitana, Ignacio Valdivieso 2409, Casilla 9845, Santiago 8940577, Chile; 2 Laboratorio de Electroquímica del Medio Ambiente, LEQMA, Departamento de Química de los Materiales, Facultad de Química y Biología, Universidad de Santiago de Chile, USACh, Casilla 40, Correo 33, Santiago 9170022, Chile; 3 Departamento de Química, Instituto de Ciencias Básicas, Universidad Técnica de Manabí, Av. Urbina y Che Guevara, Portoviejo 130104, Ecuador; oramirez@ciq.uchile.cl; 4 Campus Río Simpson, Universidad de Aysén, Obispo Vielmo 62, Coyhaique 5952039, Chile; 5 Departamento de Química, Facultad de Ciencias, Universidad de Chile, Las Palmeras 3425, Casilla 653, Santiago 7800003, Chile; bweiss@uchile.cl; 6 Instituto de Química de Recursos Naturales, Universidad de Talca, Av. Lircay s/n, Casilla 747, Talca 3460000, Chile

**Keywords:** quinones, redox potential, density functional theory, semiquinone, cyclic voltammetry

## Abstract

The rational design of quinones with specific redox properties is an issue of great interest because of their applications in pharmaceutical and material sciences. In this work, the electrochemical behavior of a series of four *p*-quinones was studied experimentally and theoretically. The first and second one-electron reduction potentials of the quinones were determined using cyclic voltammetry and correlated with those calculated by density functional theory (DFT) using three different functionals, BHandHLYP, M06-2x and PBE0. The differences among the experimental reduction potentials were explained in terms of structural effects on the stabilities of the formed species. DFT calculations accurately reproduced the first one-electron experimental reduction potentials with *R*^2^ higher than 0.94. The BHandHLYP functional presented the best fit to the experimental values (*R*^2^ = 0.957), followed by M06-2x (*R*^2^ = 0.947) and PBE0 (*R*^2^ = 0.942).

## 1. Introduction

The quinone/semiquinone/hydroquinone (Q/SQ^•−^/HQ) triad, ubiquitous in nature, is an important part of many redox systems in biology, acting as a vital link in electron transfer processes through both mitochondria and chloroplasts [1]. However, the redox couple Q/HQ can also directly interconvert through other enzymatic systems via a two-electron process [2]. An important characteristic of quinones is their capability to undergo reversible oxidation-reduction reactions [3]. Other important biological process where the Q/HQ couple is present include antioxidant metabolism (e.g., ubiquinone and tocopherol) [4], protein post-translational modification (e.g., topaquinone) [5] and blood clotting (e.g., vitamin K) [6].

Besides the importance of the Q/HQ couple as anticancer agents [7,8,9,10] antifungal [11,12], and antiparasitic drugs [13], the quinone motif is also found in many dangerous xenobiotics, such as carcinogenic polyaromatic quinones generated in air-suspended particulate by oxidation of polycyclic aromatic hydrocarbons [14]. During the last years, Q/HQ derivatives have also gained growing interest in the development of new organic materials, in areas such as solar energy conversion, [15] organic switches for molecular electronics, [16] organic light-emitting diodes (OLEDs) [16,17] and batteries [18,19,20,21]. In all of these cases, the transfer of electrons in the redox Q/HQ couple plays a central role.

Several electrochemical studies have been carried out regarding the reduction of quinones, mainly to gain insights about the underlying mechanisms and the effects provoked by changing conditions such as electrolytic medium and electrode surface, among others [22]. A study on the effects of hydrogen bonds in different Q/HQ systems in non-aqueous media showed that the presence of oxygenated functional groups on the surface of the electrode could affect the interaction between dissolved compounds and the surface, which had a significant impact on the electron transfer process [23]. The electrochemistry of quinones in the presence of added hydroquinone as a hydrogen bonding proton donor, the effective pH at the electrode surface [24] as well as the electrochemical effect of CO_2_ in dimethylformamide solution, were all studied [25].

Redox potentials indicate the tendency of a molecule to accept or donate electrons and constitute key parameters to understand electron-transfer reactions, for instance, in the Q/HQ couple [23,26,27] and in the reaction of quinones with nucleophiles via Michael addition [3]. In aqueous media, *p*-quinones undergo a reversible two-electron reduction, being the reduction potential dependent on pH [28]. In neutral aprotic media, quinones (Q) undergo a first one-electron reduction step to produce the semiquinone (SQ^•−^) radical anion, followed by a second one-electron reduction process that yields the dianion hydroquinone (HQ^−2^) [29,30].

Consequently, the determination of redox potentials of quinones is currently an issue of great interest. The rational design of quinones with specific redox properties offers a wide variety of applications in pharmaceutical and materials sciences [27,31,32,33].

The prediction of accurate redox potentials is a main issue in all areas where electron transfer reactions are important [34]. Computational chemistry is a powerful tool in electrochemistry since it can predict redox potentials prior to the synthesis of the compounds [25,35,36,37]. One of the most common approaches to calculate redox potentials is the use of a thermodynamic cycle that involves the gas phase energies and free energies of solvation [38,39,40,41,42]. On the other hand, the use of implicit solvation models has been very instructive to assess the contribution from the solvent [43,44], avoiding the use of explicit models that are commonly unpractical as a result of their computational cost.

**Figure 1:**
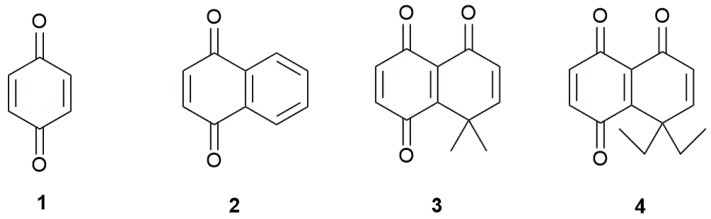
Quinones studied in this work.

In this work, we studied the electrochemical reduction of two benzoquinones bearing vicinal carbonyl and alkyl groups linked by a double bond (Figure 1). These compounds differ only by the volume of the *gem*-dialkyl group linked to the quinone ring, allowing the study of the steric effect on the reduction potentials. The electrochemical parameters of these compounds were also compared with those obtained for benzo- and naphthoquinone. The electrochemical response of these quinones on a hanging mercury drop electrode (HMDE) was studied by cyclic voltammetry. The first and second one-electron reduction potentials were calculated through density functional theory (DFT) calculations, using three different functionals, BHandHLYP, M06-2x and PBE0, to compare the results with the experimentally measured values.

## 2. Materials and Methods

### 2.1. Cyclic Voltammetry

Electrochemistry experiments were carried out in acetonitrile with tetrabutylammonium hexafluorophosphate (TBFP) as supporting electrolyte, using a hanging mercury drop electrode as the working electrode. A platinum wire counter-electrode and a non-aqueous Ag/AgCl_(sat)_ (CHInstrument, Austin, TX, USA) electrode were used as a reference for the measurements. The concentration of the solution was 0.5 mM for each compound. In all experiments, the resistance was automatically compensated.

### 2.2. Computational Details

The calculations were carried out using the Gaussian 09 [45] program package (Gaussian, Inc., Wallingford, CT, USA) running in a Microsystem (Sun Microsystem, Menlo Park, CA, USA) cluster of blades. Geometries were calculated at the DFT BHandHLYP, M06-2x and PBE0 level, using the 6-31+G(d,p) basis set. The conductor-like polarizable continuum model (C-PCM) approach was used to include the role of the solvent, acetonitrile in this case. No imaginary vibrational frequencies were found at the optimized geometries, which indicate that they are true minima of the potential energy surface. Cartesian coordinates and energies for the calculated optimized structures can be found in the Supplementary material.

**Scheme 1:**
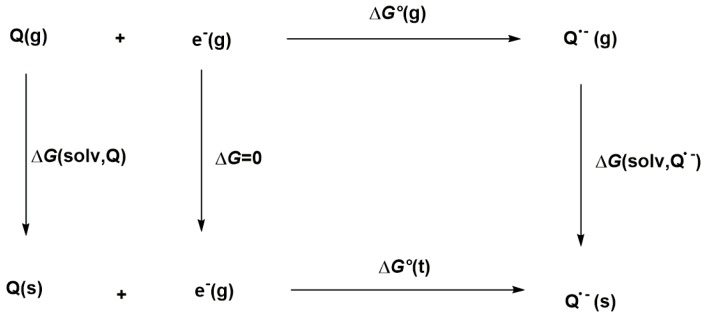
Thermodynamic cycle used to calculate the Gibbs free energy (Δ*G*°(t)).

The thermodynamic cycle displayed in Scheme 1 was used to calculate the Gibbs free energy of the reaction in solution, Δ*G*°(t).

From Δ*G*°(t), the reduction potential *E*°_red_ with respect to Ag/AgCl was obtained by Equation (1):

(1) $$E_{red}^{0}=\frac{-\Delta G^{0}\left(t\right)}{nF}-4.72V$$

Here, the constant 4.72 V corresponds to the redox potential of the Ag/AgCl couple, the reference electrode.

## 3. Results and Discussion

### 3.1. Cyclic Voltammetry

**Figure 2:**
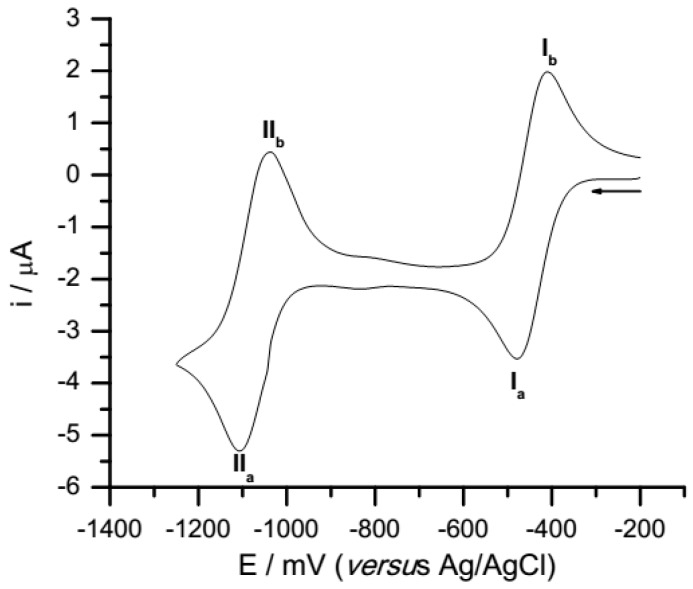
Cyclic voltammograms of quinone 1, 0.5 mM in acetonitrile +0.1 M tetrabutylammonium hexafluorophosphate at 100 mVs^−1^.

Voltammograms of all compounds display two well defined peaks (Figure 2). The values presented in Table 1 correspond to the peak potential averages from three independent voltamogramms, all measured at a sweep rate of 100 mV/s. The first process can be associated with the addition of one electron to the quinone species to form the semiquinone anion radical (SQ^●^^−^), and the second wave could be associated with the addition of another electron to form the hydroquinone di-anion (HQ^−2^).

Table 1 presents parameters obtained from the voltammetric curves. ΔEp^I^ values (difference between peak potential Ep^Ia^-Ep^Ib^), between −79 and −65 mV, are indicative of a reversible process for the first mono-electronic couple, in all quinones. The ΔEp^II^ values (difference between peak potential Ep^IIa^-Ep^IIb^), for the second mono-electronic reduction process, indicate a reversible process for quinones **1** and **2**; however, the increasing absolute values for **3** and **4** are indicative of quasi-reversible processes in these two latter cases.

*p*-Naphtoquinone (**2**) presents the most negative reduction potential for the first mono-electronic reduction, followed by *p*-benzoquinone (**1**). As expected, quinones **3** and **4** exhibit very close values for both reduction potentials (7 mV difference, while the standard deviations of the peak potential are between +1 and +9 mV). For the second mono-electronic reduction, *p*-benzoquinone (**1**) is the one that presents the most negative potential, followed by *p*-naphtoquinone (**2**), and then quinones **4** and **3**. Quinones **1**–**2** and **3**–**4** exchange positions in the second reduction potential rank with respect to the first reduction potential. The presence of the fused aromatic ring moiety in *p*-naphtoquinone (**2**) decreases the spontaneity for the formation of the semi-quinone radical, displacing the first reduction potential to a more negative value. On the other hand, the presence of the aromatic moiety in *p*-naphtoquinone (**2**) favors the reduction of the semiquinone to hydroquinone dianion, when compared with *p*-benzoquinone (**1**), while the presence of an *o*-carbonyl group (quinones **3** and **4**) favors the second reduction process of these molecules, even more than the presence of the aromatic moiety. The electrochemical parameters of both quinones **1** and **2** are close to previously reported values [46]. Table 2 presents the values *ΔE°_I–II_ = E°_I_ − E°_II_*, which allow us to calculate the equilibrium constant for the comproportionation reaction [47,48] from *ln K = F/RT × (ΔE_I–II_)*.

The equilibrium constant for the comproportionation reaction allows us to discuss the thermodynamic stability of the radical anion semiquinone. High values of *K* indicate that the formed radical anion is more stable. From Table 2, we observe that *p*-naphtoquinone (**2**) generates the less stable radical. This unusual behavior of aromatic quinones, where an increase in conjugated aromatic rings leads to a reduction potential decrease, diminishing spontaneity, is opposite to the usual behavior observed and reported for most organic aromatic molecules [49]. The thermodynamic stability of the semi-quinone radical generated from quinones **3** and **4** is more closely related to *p*-benzoquinone.

On the other hand, quinone **4** presents the same structure as quinone **3**, except that the *gem*-dimethyl group was replaced by a *gem*-diethyl group, allowing us to evaluate the effect of sterical shielding on the redox properties of this type of quinone. The more negative second reduction potential of quinone **3** compared with **4,** shows a steric effect different from that previously reported for imidazoline nitroxides, [50] for which a difference of 16 mV has been reported for the replacement of a *gem*-dimethyl group for a *gem*-diethyl moiety, with the more bulky substituents exhibiting the most negative reduction potential.

The semiquinone derived from **4** shows a radical thermodynamic stability higher than the semiquinone from **3** and from **1**. This result suggests that the higher volume of *gem*-diethyl groups and their conformation, displaying the methyl groups of the ethyl moieties above and below the molecular plane [51], is a structural factor that provided more thermodynamic stability to the generated semiquinone radical.

### 3.2. Theoretical First and Second One-Electron Reduction Potentials

The first and second one-electron reduction potentials were calculated for the four quinones at the DFT level using three different functionals BHandHLYP, M06-2x and PBE0, which have been suggested as useful for this kind of calculation [27,52]. These functionals were employed together with a 6-31+G(d,p) basis set. The solvent effect was considered through a continuum model of solvation, C-PCM, which has been successfully used for calculations of quinone reduction potentials [53,54]. The results of the first and second one-electron reduction potentials are presented in Table 3.

**Figure 3:**
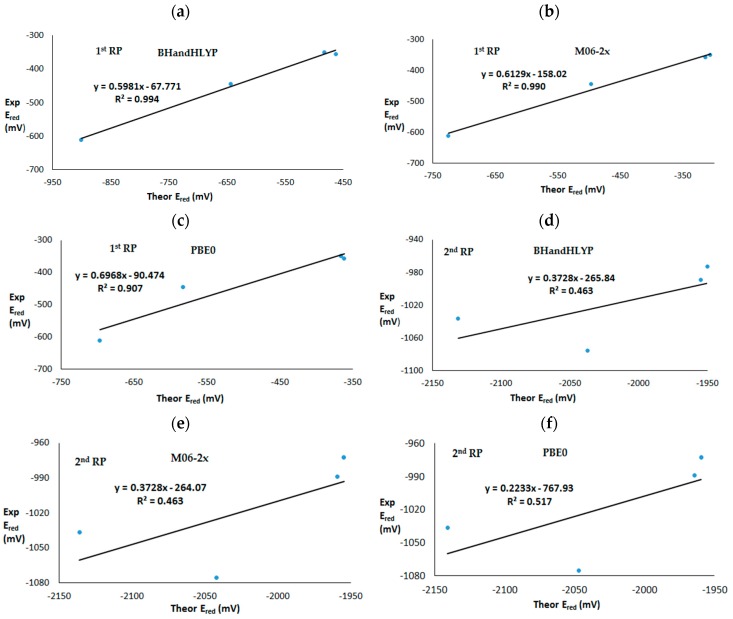
Experimental versus theoretical first and second one-electron reduction potentials. First reduction potential at (**a**) BhandHLYP, (**b**) M06-2x and (**c**) PBE0 level. Second reduction potential at (**d**) BhandHLYP, (**e**) M06-2x and (**f**) PBE0 level.

Among the four studied quinones, *p*-naphtoquinone (**2**) presents the most deviated theoretical value with respect to the experimental ones, in all cases except for the one-electron reduction potential at the PBE0 level where *p*-benzoquinone (**1**) also presents a value far removed from the experimental one. The correlations among experimental and calculated first and second one-electron reduction potentials at different DFT levels are presented in Figure 3. A recent study dealing with calculations of aqueos redox potentials for 19 organic compounds using a wide range of levels (ab-initio and DFT) found that M06-2x was the only DFT method, among all studied, that predicted potentials at a similar level of accuracy than the local pair natural orbital (LPNO), which yielded close results when compared with coupled cluster including single and double excitations with perturbative triples, CCSD(T) [34].

In our case, calculated first reduction potentials at different DFT functional levels present good correlations with experimental values. The BHandHLYP level exhibited the best fit to the experimental potentials, showing *R*^2^ = 0.994, followed by M06-2x with *R*^2^ = 0.990 and PBE0 with *R*^2^ = 0.907. However, the calculated second mono-electron reduction potentials did not present a good correlation with experimental values. Calculated second reduction potentials had *R*^2^ = 0.462 at the BHandHLYP and M06-2x levels and *R*^2^ = 0.517 at the PBE0 level. In previous work, the quality of the calculated second reduction potential of quinones was shown to be poor, which can be associated with the difficult treatment of the dianion species [55]. Specifically, the ion pair formation [56] between the anion species and the cation of support electrolite tetabutilammonium (TBA^+^) can exert an influence on the formation of dianion species [57]. Some studies have examined this subject, showing that when ion pair formation is favourable, stabilizing the dianion species, the second reduction potential can increase [58,59].

We also obtained the highest occupied molecular orbital (HOMO) and the lowest unoccupied molecular orbital (LUMO) energies for neutral quinones, semiquinone radicals and hydroquinone dianions (Table 4). The differences in LUMO energies suggest a plausible explanation for the observed reduction potential for quinones **3** and **4**. Neutral species of **4** presented slightly higher LUMO energy values (−2.66 eV) compared with **3** (−2.69 eV), which can explain the close potential for the first reduction in both molecules (−357 and 350 mV for **3** and **4**, respectively). By contrast, semiquinone derived from **3** presented a higher LUMO energy (2.23 eV) compared with the semiquinone species of **4** (2.19 eV), which can explain the easier reduction of **3** compared with **4** (E° −973 mV and −989 mV, respectively).

## 4. Conclusions

In this work, the reduction processes of four quinones in acetonitrile were studied experimentally, and the results were compared with those calculated through DFT methods. *p*-Naphtoquinone (**2**) generated a less stable radical than *p*-benzoquinone (**1**), and the thermodynamic stability of radicals generated from quinones **3** and **4**, which are more closely related to those from *p*-benzoquinone (**1**), showed that the bulkier substituents led to a more negative value for both first and second one-electron reduction potentials. The presence of a *o*-carbonyl group in quinones **3** and **4** decreased the thermodynamic stability of the semiquinone radicals.

The first one-electron reduction potentials calculated at the DFT level using three different functionals, i.e., BHandHLYP, M06-2x and PBE0, accurately reproduced the experimental potentials with *R*^2^ higher than 0.94 in all cases. The BHandHLYP level represented the best fit with experimental potential (*R*^2^ = 0.994), followed by M06-2x (*R*^2^ = 0.990) and PBE0 (*R*^2^ = 0.907). However, the calculated second reduction potentials were not well correlated with experimental values. The LUMO energies for neutral quinones, semiquinone radicals and hydroquinone dianions lead us to rationalize the observed difference in reduction potential among these quinones.

## Figures and Tables

**Table 1:** Parameters from voltammetric curves: Anodic (Epa) and catodic (Epc) peak potentials (±SD). Formal reduction potential E°_I_ (Q → SQ^•−^) and E°_II_ (SQ^•−^ → HQ^2−^) and *ΔEp = Epc − Epa*. All values are in mV.

Compounds	Semiquinone		Hidroquinone Dianion		E°_I_	E°_II_	ΔEp^I^	ΔEp^II^
	Epc^Ia^	Epa^Ib^	Epc^IIa^	Epa^IIb^				
**1**	−479 ± 2	−410 ± 6	−1107 ± 4	−1044 ± 4	−445	−1076	−69	−63
**2**	−650 ± 3	−571 ± 5	−1066 ± 4	−1007 ± 2	−611	−1037	−79	−59
**3**	−390 ± 1	−323 ± 9	−1022 ± 4	−923 ± 5	−357	−973	−67	−99
**4**	−382 ± 2	−317 ± 5	−1033 ± 3	−945 ± 3	−350	−989	−65	−88

**Table 2:** Values *ΔE°_I–II_ = E°_I_ − E°_II_* and the equilibrium constant for the comproportionation reaction.

Compounds	ΔE°_I–II_ (mV)	ln *K*
**1**	631	25.2
**2**	426	17.0
**3**	616	24.6
**4**	639	25.6

**Table 3:** Experimental and calculated first and second reduction potentials (Ered). All values are in mV.

	Compound	Ered			
		BHandHLYP	M06	PBE0	E°(exp)
**1st RP**	**1**	−644	−497	−583	−445
	**2**	−901	−725	−697	−611
	**3**	−463	−314	−362	−357
	**4**	−483	−306	−366	−350
**2nd RP**	**1**	−2037	−1769	−1843	−1076
	**2**	−2131	−1842	−1934	−1037
	**3**	−1950	−1651	−1624	−973
	**4**	−1954	−1669	−1660	−989

RP, reduction potential.

**Table 4:** Highest occupied molecuar orbital (HOMO) and lowest unoccupied molecular orbital (LUMO) energies (eV) for neutral quinone (Q), the semiquinone radical (SQ^•−^) and hydroquinone dianion (HQ**^−2^**) at the BHandHLYP/6–31 + G(d,p) level.

Molecule	Q		SQ^•−^		HQ^−2^	
	E_HOMO_	E_LUMO_	E_SOMO_	E_LUMO_	E_HOMO_	E_LUMO_
**1**	−9.58	−2.59	−1.37	2.98	3.99	7.36
**2**	−8.79	−2.28	−1.31	2.72	3.58	5.94
**3**	−8.55	−2.69	−1.87	2.23	2.93	5.71
**4**	−8.51	−2.66	−1.91	2.19	2.82	5.48

## Supplementary Materials

Supplementary materials are available online.
