# Supplementary material for: Experimental and Theoretical Reduction Potentials of Some Biologically Active ortho-Carbonyl para-Quinones
Source: Molecules. 2017 Apr 4;22(4):577. doi: 10.3390/molecules22040577 (PMC6154728; doi:10.3390/molecules22040577)
Supplement: Supplementary file 1 [file molecules-22-00577-s001.pdf]

SUPPLEMENTARY INFORMATION

Experimental and Theoretical One- and Two-Electron Reduction Potentials of Some Biologically Active *Ortho*-carbonyl Quinones

Maximiliano Martínez-Cifuentes[1*], Ricardo Salazar[2*], Oney Ramirez-Rodriguez[3,4], Boris Weiss-Lopéz[4], Ramiro Araya-Maturana[5*]

1. Programa Institucional de Fomento a la Investigación, Desarrollo e Innovación, Universidad Tecnológica Metropolitana, Ignacio Valdivieso 2409, Casilla 9845, Santiago 8940577, Chile.
2. Laboratorio de Electroquímica del Medio Ambiente, LEQMA, Departamento de Química de los Materiales, Facultad de Química y Biología, Universidad de Santiago de Chile, USACh, Casilla 40, Correo 33, Santiago 9170022, Chile.
3. Departamento de Química, Instituto de Ciencias Básicas, Universidad Técnica de Manabí, Av. Urbina y Che Guevara, Portoviejo, Ecuador.
4. Campus Río Simpson, Universidad de Aysén, Obispo Vielmo 62, Coyhaique, Chile.
5. Departamento de Química, Facultad de Ciencias, Universidad de Chile, Las Palmeras 3425, Casilla 653, Santiago 7800003, Chile.
6. Instituto de Química de Recursos Naturales, Universidad de Talca, Av. Lircay s/n, Casilla 747, Talca 3460000, Chile

* Correspondences: mmartinez@utem.cl (M.M.-C.); raraya@utalca.cl (R.A.-M.); ricardo.salazar@usach.cl (R.S.)
Tel.: +56-22-787-7902 (M.M.-C); +56-71-220-0285 (R.A.-M.); +56-22-718-1178 (R.S.)

1. Optimized geometries

1.1 Neutral

1.1.1 BHandHLYP/6-31+G(d,p) level

Q1

6	0.663436	1.264348	0.000041
6	-0.663436	1.264348	0.000038
6	-1.432750	0.000000	-0.000555
6	-0.663436	-1.264348	0.000038
6	0.663436	-1.264348	0.000041
6	1.432750	0.000000	-0.000565
1	1.246198	2.169566	0.000378
1	-1.246198	2.169567	0.000369
1	-1.246198	-2.169566	0.000369
1	1.246198	-2.169567	0.000378
8	2.635205	0.000000	0.000284
8	-2.635205	0.000000	0.000251

Q2

6	-2.657659	-0.693718	0.000022
6	-1.463351	-1.388849	0.000023
6	-0.261859	-0.697176	0.000006
6	-0.261859	0.697176	-0.000040
6	-1.463351	1.388849	-0.000028
6	-2.657659	0.693718	0.000000
1	-3.590223	-1.230910	0.000045
1	-1.441790	-2.463931	0.000056

6	1.017651	-1.451115	0.000064
6	1.017651	1.451115	-0.000136
1	-1.441790	2.463931	-0.000063
1	-3.590223	1.230909	-0.000002
6	2.269214	0.663609	-0.000005
6	2.269214	-0.663608	0.000022
1	3.178712	1.239913	0.000024
1	3.178712	-1.239913	0.000049
8	1.053666	2.653806	0.000089
8	1.053666	-2.653806	-0.000049

Q3

6	1.058279	-2.494682	-0.000041
6	-0.257554	-1.840692	0.000006
6	-0.322363	-0.336358	0.000022
6	0.819914	0.378455	0.000033
6	2.155851	-0.306929	0.000038
6	2.173410	-1.785652	-0.000019
1	1.040464	-3.570793	-0.000089
6	0.789581	1.880582	0.000036
1	3.153810	-2.229954	-0.000042
6	-0.538274	2.496975	-0.000013
6	-1.646557	1.776700	-0.000020
1	-0.548237	3.573062	-0.000037
1	-2.606989	2.269516	-0.000045
8	3.199554	0.280066	0.000055
8	-1.253784	-2.516634	-0.000089
8	1.780598	2.557069	-0.000012
6	-1.705714	0.284518	0.000008
6	-2.489562	-0.132476	-1.264645
1	-2.673017	-1.197138	-1.276914
1	-3.445678	0.382078	-1.276596
1	-1.948597	0.147980	-2.163005
6	-2.489573	-0.132421	1.264674
1	-3.445681	0.382149	1.276603
1	-2.673043	-1.197079	1.276981
1	-1.948606	0.148060	2.163026

Q4

6	-1.363969	-2.474157	-0.000196
6	-0.030242	-1.856933	0.000040
6	0.078452	-0.356109	0.000065
6	-1.043576	0.390324	0.000072
6	-2.398226	-0.256106	0.000253
6	-2.458552	-1.733750	-0.000110
1	-1.376496	-3.550381	-0.000400
6	-0.970886	1.891030	-0.000102
1	-3.451340	-2.149718	-0.000217
6	0.373750	2.470064	-0.000050
6	1.459631	1.716735	0.000004
1	0.414606	3.545602	-0.000110
1	2.434123	2.182162	0.000012
8	-3.425401	0.359881	0.000468
8	0.946281	-2.561652	-0.000172
8	-1.942626	2.595515	-0.000410
6	1.478512	0.224108	0.000039
6	2.276927	-0.225068	1.259295
1	2.491582	-1.281448	1.168287

1	3.231914	0.294473	1.220557
6	2.276911	-0.225139	-1.259201
1	3.231905	0.294390	-1.220497
1	2.491554	-1.281518	-1.168138
6	1.602351	0.055916	2.593349
1	2.261054	-0.225597	3.408841
1	1.357552	1.106795	2.713086
1	0.684250	-0.512830	2.708985
6	1.602329	0.055783	-2.593264
1	1.357531	1.106658	-2.713047
1	2.261027	-0.225768	-3.408746
1	0.684226	-0.512966	-2.708870

1.1.2 M06-2x/6-31+G(d,p) level

Q1

6	0.666830	1.272620	-0.000001
6	-0.666830	1.272620	-0.000022
6	-1.438972	0.000000	-0.000162
6	-0.666830	-1.272620	-0.000022
6	0.666830	-1.272620	-0.000001
6	1.438972	0.000000	-0.000053
1	1.256882	2.182307	0.000061
1	-1.256882	2.182307	0.000037
1	-1.256883	-2.182306	0.000037
1	1.256882	-2.182307	0.000061
8	2.648291	0.000000	0.000058
8	-2.648291	0.000000	0.000113

Q2

6	-2.669361	-0.697268	0.000021
6	-1.469574	-1.396410	0.000027
6	-0.264185	-0.699594	0.000009
6	-0.264185	0.699594	-0.000034
6	-1.469574	1.396410	-0.000031
6	-2.669361	0.697268	-0.000005
1	-3.608340	-1.237697	0.000044
1	-1.444247	-2.479528	0.000059
6	1.022369	-1.458127	0.000060
6	1.022369	1.458128	-0.000114
1	-1.444247	2.479528	-0.000065
1	-3.608340	1.237696	-0.000010
6	2.282387	0.667121	-0.000003
6	2.282387	-0.667121	0.000017
1	3.196786	1.250245	0.000021
1	3.196786	-1.250245	0.000038
8	1.055748	2.667408	0.000080
8	1.055748	-2.667408	-0.000050

Q3

6	-1.039587	-2.516261	-0.000008
6	0.278505	-1.845432	0.000000
6	0.328510	-0.333151	0.000006
6	-0.825558	0.374273	0.000011
6	-2.161527	-0.324485	0.000004
6	-2.166873	-1.812897	-0.000002
1	-1.009661	-3.599828	-0.000019
6	-0.809325	1.883589	0.000020

1	-3.151664	-2.266572	-0.000007
6	0.522952	2.513420	-0.000003
6	1.641194	1.794464	-0.000010
1	0.524605	3.596959	-0.000011
1	2.608063	2.293253	-0.000021
8	-3.215095	0.259625	-0.000005
8	1.287616	-2.515424	-0.000034
8	-1.812254	2.555179	0.000020
6	1.707552	0.297423	0.000002
6	2.494234	-0.115528	1.268660
1	2.699324	-1.183688	1.272188
1	3.445027	0.422447	1.286270
1	1.935996	0.152265	2.168421
6	2.494230	-0.115550	-1.268652
1	3.445027	0.422417	-1.286269
1	2.699312	-1.183712	-1.272164
1	1.935993	0.152235	-2.168416

Q4

6	-1.297345	-2.506992	-0.000081
6	0.029482	-1.853563	-0.000011
6	0.101212	-0.344136	0.000006
6	-1.041921	0.380248	-0.000010
6	-2.387207	-0.298444	0.000098
6	-2.414286	-1.786934	-0.000044
1	-1.283227	-3.590906	-0.000137
6	-1.001513	1.888621	-0.000139
1	-3.405735	-2.225962	-0.000048
6	0.340969	2.497233	-0.000040
6	1.446046	1.757976	0.000019
1	0.360904	3.580817	-0.000063
1	2.421268	2.241319	0.000062
8	-3.433097	0.299837	0.000438
8	1.030909	-2.535876	0.000012
8	-1.993508	2.576887	-0.000383
6	1.490045	0.260395	0.000016
6	2.287376	-0.173518	1.268092
1	2.569792	-1.220236	1.159513
1	3.213130	0.411038	1.270988
6	2.287406	-0.173520	-1.268040
1	3.213154	0.411046	-1.270927
1	2.569831	-1.220235	-1.159446
6	1.534084	0.038927	2.578001
1	2.184007	-0.170707	3.429014
1	1.172888	1.066014	2.673031
1	0.670797	-0.627672	2.652389
6	1.534134	0.038902	-2.577965
1	1.172924	1.065983	-2.673011
1	2.184078	-0.170728	-3.428963
1	0.670860	-0.627712	-2.652363

1.1.3 PBE0/6-31+G(d,p) level

Q1

6	-0.668533	1.264687	-0.000004
6	0.668533	1.264687	0.000017
6	1.437718	0.000000	0.000069
6	0.668533	-1.264687	0.000017

6	-0.668533	-1.264687	-0.000004
6	-1.437718	0.000000	-0.000047
1	-1.255703	2.177715	-0.000010
1	1.255703	2.177716	0.000015
1	1.255704	-2.177715	0.000015
1	-1.255703	-2.177715	-0.000010
8	-2.652792	0.000000	0.000011
8	2.652792	0.000000	-0.000049

Q2

6	2.665683	-0.696837	0.000178
6	1.466379	-1.394822	-0.000061
6	0.259192	-0.700774	-0.000285
6	0.259192	0.700775	-0.000244
6	1.466379	1.394822	-0.000015
6	2.665683	0.696837	0.000198
1	3.605935	-1.238268	0.000339
1	1.440503	-2.479123	-0.000112
6	-1.020632	-1.456946	-0.000655
6	-1.020632	1.456946	-0.000474
1	1.440503	2.479123	-0.000006
1	3.605935	1.238269	0.000383
6	-2.272292	0.668786	0.000216
6	-2.272292	-0.668786	0.000192
1	-3.189933	1.249211	0.000695
1	-3.189932	-1.249212	0.000673
8	-1.055811	2.671689	0.000170
8	-1.055810	-2.671689	0.000296

Q3

6	-0.994679	-2.522272	-0.000009
6	0.305807	-1.837543	0.000002
6	0.334353	-0.332340	0.000008
6	-0.833066	0.363667	0.000013
6	-2.152692	-0.354650	0.000002
6	-2.134875	-1.834128	-0.000003
1	-0.951918	-3.606755	-0.000022
6	-0.837165	1.868069	0.000028
1	-3.112576	-2.305527	-0.000010
6	0.478836	2.511888	-0.000003
6	1.611401	1.811307	-0.000012
1	0.466660	3.596834	-0.000013
1	2.570304	2.327108	-0.000026
8	-3.221154	0.212964	-0.000010
8	1.327852	-2.497919	-0.000039
8	-1.852413	2.529729	0.000025
6	1.700098	0.321080	0.000002
6	2.494950	-0.078949	1.266078
1	2.700866	-1.148474	1.275070
1	3.447083	0.459699	1.275554
1	1.944946	0.192438	2.171141
6	2.494946	-0.078975	-1.266069
1	3.447082	0.459667	-1.275556
1	2.700854	-1.148502	-1.275042
1	1.944942	0.192400	-2.171135

Q4

6	-1.324163	2.496083	0.000653

6	0.001703	1.861177	0.000746
6	0.089161	0.359304	0.000277
6	-1.051184	-0.380794	0.000130
6	-2.397280	0.285407	0.000273
6	-2.437007	1.764521	0.000497
1	-1.323105	3.581436	0.000714
6	-0.997138	-1.884204	-0.000210
1	-3.432358	2.197509	0.000421
6	0.342805	-2.477206	-0.000897
6	1.445710	-1.730736	-0.000790
1	0.373330	-3.561992	-0.001397
1	2.424891	-2.208317	-0.001194
8	-3.443775	-0.322221	-0.000426
8	0.997407	2.561239	0.000223
8	-1.986075	-2.585090	-0.000327
6	1.477802	-0.239507	-0.000065
6	2.281353	0.200638	-1.262165
1	2.507299	1.263932	-1.166611
1	3.238521	-0.333005	-1.221335
6	2.281518	0.199366	1.262376
1	3.238613	-0.334364	1.220970
1	2.507591	1.262716	1.167799
6	1.593836	-0.077545	-2.590325
1	2.251709	0.193521	-3.420249
1	1.332527	-1.133789	-2.703103
1	0.674171	0.505108	-2.700572
6	1.594077	-0.079973	2.590332
1	1.332649	-1.136291	2.702150
1	2.252050	0.190242	3.420454
1	0.674491	0.502688	2.701198

1.2 Semiquinone radical anion

1.2.1 BHandHLYP/6-31+G(d,p) level

Q1

6	-0.000002	0.679685	1.213825
6	-0.000001	-0.679685	1.213825
6	0.000003	-1.454881	0.000000
6	-0.000001	-0.679685	-1.213825
6	-0.000002	0.679685	-1.213825
6	-0.000002	1.454881	0.000000
1	-0.000003	1.234326	2.139448
1	-0.000003	-1.234326	2.139448
1	-0.000003	-1.234326	-2.139448
1	-0.000003	1.234326	-2.139448
8	0.000007	2.706401	0.000000
8	-0.000002	-2.706401	0.000000

Q2

6	-2.639362	-0.700188	0.000014
6	-1.449644	-1.384834	-0.000034
6	-0.225641	-0.703645	-0.000061
6	-0.225641	0.703645	-0.000004
6	-1.449644	1.384834	0.000029
6	-2.639362	0.700188	0.000037
1	-3.573714	-1.238558	0.000018

1	-1.418576	-2.460509	-0.000094
6	1.027533	-1.467324	-0.000227
6	1.027533	1.467324	0.000093
1	-1.418576	2.460509	0.000084
1	-3.573714	1.238558	0.000077
6	2.219237	0.684917	0.000042
6	2.219237	-0.684917	-0.000014
1	3.148693	1.232127	0.000104
1	3.148693	-1.232128	0.000045
8	1.031358	2.714444	-0.000277
8	1.031357	-2.714444	0.000341

Q3

6	-0.795303	2.523642	-0.000075
6	0.443333	1.789465	-0.000193
6	0.348414	0.346151	-0.000005
6	-0.892452	-0.293538	0.000055
6	-2.138887	0.475685	-0.000099
6	-1.993518	1.917768	0.000007
1	-0.702577	3.598127	-0.000066
6	-0.970292	-1.766965	0.000271
1	-2.915032	2.477106	0.000100
6	0.309063	-2.502932	0.000013
6	1.481507	-1.896872	-0.000040
1	0.214779	-3.576555	-0.000064
1	2.393403	-2.478912	-0.000128
8	-3.270718	-0.013551	0.000270
8	1.533660	2.409840	-0.000297
8	-1.992642	-2.418562	-0.000082
6	1.668838	-0.416599	0.000028
6	2.493346	-0.074861	-1.259073
1	2.721538	0.981671	-1.277663
1	3.422439	-0.643501	-1.259092
1	1.937681	-0.336639	-2.154971
6	2.493279	-0.074977	1.259206
1	3.422350	-0.643653	1.259242
1	2.721505	0.981544	1.277892
1	1.937547	-0.336805	2.155049

Q4

6	-1.198530	2.491261	0.000398
6	0.086295	1.840192	0.000214
6	0.089403	0.394732	0.000092
6	-1.106647	-0.325483	0.000006
6	-2.401986	0.358663	-0.000130
6	-2.353727	1.807538	0.000253
1	-1.176572	3.569527	0.000631
6	-1.087878	-1.801157	0.000102
1	-3.310624	2.303921	0.000354
6	0.236776	-2.451930	-0.000525
6	1.365549	-1.768644	-0.000489
1	0.213777	-3.529595	-0.000871
1	2.313864	-2.289985	-0.000789
8	-3.498528	-0.204586	-0.000202
8	1.131996	2.533627	0.000379
8	-2.065791	-2.517835	-0.000237
6	1.457802	-0.279053	-0.000037
6	2.286241	0.123973	-1.251659

1	2.512208	1.178818	-1.168063
1	3.230511	-0.420638	-1.204043
6	2.286194	0.123232	1.251856
1	3.230452	-0.421376	1.203970
1	2.512189	1.178119	1.168880
6	1.611195	-0.151793	-2.586881
1	2.269032	0.128095	-3.406800
1	1.356738	-1.201584	-2.705442
1	0.695512	0.422090	-2.685050
6	1.611082	-0.153291	2.586889
1	1.356579	-1.203141	2.704825
1	2.268896	0.126091	3.406998
1	0.695419	0.420572	2.685356

1.2.2 M06-2x/6-31+G(d,p) level

Q1

6	0.000000	-0.682926	1.221506
6	0.000000	0.682926	1.221506
6	0.000003	1.463204	0.000000
6	0.000000	0.682926	-1.221506
6	0.000000	-0.682926	-1.221506
6	-0.000023	-1.463204	0.000000
1	0.000009	-1.245208	2.151090
1	0.000004	1.245208	2.151090
1	0.000004	1.245208	-2.151090
1	0.000009	-1.245208	-2.151090
8	0.000057	-2.720547	0.000000
8	-0.000045	2.720547	0.000000

Q2

6	-2.651117	-0.703575	0.000015
6	-1.455073	-1.391897	-0.000053
6	-0.227301	-0.706504	-0.000083
6	-0.227301	0.706505	-0.000004
6	-1.455073	1.391897	0.000046
6	-2.651117	0.703575	0.000055
1	-3.591467	-1.245314	0.000020
1	-1.420352	-2.475611	-0.000132
6	1.031415	-1.476591	-0.000288
6	1.031415	1.476591	0.000108
1	-1.420351	2.475611	0.000119
1	-3.591467	1.245314	0.000110
6	2.231113	0.687824	0.000056
6	2.231113	-0.687824	-0.000018
1	3.165001	1.241978	0.000143
1	3.165001	-1.241978	0.000056
8	1.034074	2.729453	-0.000350
8	1.034074	-2.729452	0.000435

Q3

6	0.778750	2.541079	-0.000008
6	-0.463116	1.794152	-0.000058
6	-0.353624	0.342172	-0.000016
6	0.895468	-0.288473	-0.000003
6	2.145945	0.490976	-0.000049
6	1.988480	1.941086	0.000004
1	0.673959	3.622064	0.000017

6	0.986937	-1.768000	0.000057
1	2.913224	2.509526	0.000043
6	-0.296052	-2.517649	0.000006
6	-1.478303	-1.913249	-0.000008
1	-0.193096	-3.597977	-0.000005
1	-2.396742	-2.500482	-0.000025
8	3.284307	0.002018	0.000069
8	-1.563693	2.408607	-0.000042
8	2.020654	-2.414071	-0.000002
6	-1.670589	-0.428769	0.000000
6	-2.496494	-0.089495	1.263049
1	-2.735833	0.972496	1.275577
1	-3.423929	-0.672982	1.265619
1	-1.926571	-0.345359	2.159737
6	-2.496522	-0.089484	-1.263027
1	-3.423958	-0.672968	-1.265582
1	-2.735858	0.972508	-1.275540
1	-1.926619	-0.345340	-2.159731

Q4

6	1.140233	2.517936	-0.000100
6	-0.139866	1.838131	-0.000206
6	-0.110123	0.383517	-0.000010
6	1.102607	-0.313816	0.000069
6	2.393986	0.396357	0.000006
6	2.315589	1.853443	0.000030
1	1.094081	3.602990	-0.000126
6	1.114129	-1.796508	0.000205
1	3.269848	2.370832	0.000137
6	-0.207508	-2.475293	0.000007
6	-1.353512	-1.805253	-0.000012
1	-0.165193	-3.559818	-0.000079
1	-2.302644	-2.342235	-0.000072
8	3.503799	-0.153396	0.000505
8	-1.206520	2.509948	-0.000314
8	2.112005	-2.496932	-0.000386
6	-1.467777	-0.311628	0.000027
6	-2.295934	0.076087	1.259435
1	-2.582674	1.122984	1.160304
1	-3.212525	-0.528080	1.247112
6	-2.295998	0.076143	-1.259318
1	-3.212607	-0.527998	-1.246965
1	-2.582705	1.123047	-1.160139
6	-1.550378	-0.137424	2.574094
1	-2.203168	0.069831	3.426228
1	-1.182586	-1.163273	2.665398
1	-0.688721	0.530929	2.636613
6	-1.550519	-0.137344	-2.574024
1	-1.182733	-1.163192	-2.665363
1	-2.203357	0.069924	-3.426117
1	-0.688866	0.531011	-2.636580

1.2.3 PBE0/6-31+G(d,p)

Q1

6	0.000000	0.683648	1.217300
6	0.000000	-0.683648	1.217300
6	0.000093	-1.463624	0.000000

6	0.000000	-0.683648	-1.217300
6	0.000000	0.683648	-1.217300
6	0.000040	1.463624	0.000000
1	-0.000040	1.243764	2.150528
1	-0.000050	-1.243764	2.150528
1	-0.000050	-1.243764	-2.150528
1	-0.000040	1.243764	-2.150528
8	0.000048	2.723585	0.000000
8	-0.000124	-2.723585	0.000000

Q2

6	2.649945	-0.702513	-0.000007
6	1.453013	-1.390083	-0.000067
6	0.225527	-0.708017	-0.000104
6	0.225527	0.708017	-0.000060
6	1.453014	1.390082	-0.000001
6	2.649945	0.702512	0.000023
1	3.591499	-1.246079	0.000011
1	1.416616	-2.475351	-0.000111
6	-1.029374	-1.476133	-0.000232
6	-1.029373	1.476134	-0.000046
1	1.416617	2.475350	0.000037
1	3.591499	1.246078	0.000072
6	-2.226227	0.687952	0.000096
6	-2.226228	-0.687951	0.000055
1	-3.162957	1.240951	0.000251
1	-3.162957	-1.240949	0.000196
8	-1.035307	2.731328	-0.000002
8	-1.035309	-2.731328	0.000202

Q3

6	0.754500	2.541984	-0.000032
6	-0.476676	1.788507	-0.000102
6	-0.357247	0.339391	-0.000012
6	0.898940	-0.285668	0.000019
6	2.139808	0.506993	-0.000082
6	1.969725	1.949675	0.000001
1	0.643751	3.623974	-0.000012
6	0.998908	-1.759386	0.000151
1	2.890850	2.527112	0.000057
6	-0.272366	-2.512476	0.000005
6	-1.462401	-1.919575	-0.000028
1	-0.162335	-3.593695	-0.000033
1	-2.376281	-2.516119	-0.000077
8	3.286567	0.030002	0.000124
8	-1.584008	2.395297	-0.000136
8	2.041137	-2.402090	0.000003
6	-1.665630	-0.441798	0.000008
6	-2.494583	-0.107108	1.260193
1	-2.727738	0.958099	1.273467
1	-3.427309	-0.685128	1.258549
1	-1.932705	-0.367022	2.162253
6	-2.494627	-0.107050	-1.260133
1	-3.427360	-0.685055	-1.258475
1	-2.727767	0.958161	-1.273355
1	-1.932787	-0.366935	-2.162225

Q4

6	1.172087	2.509332	-0.000299
6	-0.110963	1.846844	-0.000208
6	-0.099084	0.393414	-0.000035
6	1.108911	-0.320733	0.000004
6	2.404557	0.378856	0.000072
6	2.340783	1.830499	-0.000160
1	1.139494	3.596496	-0.000460
6	1.102571	-1.798283	-0.000079
1	3.301740	2.338860	-0.000179
6	-0.219391	-2.458119	0.000262
6	-1.362190	-1.778928	0.000303
1	-0.188250	-3.544659	0.000386
1	-2.317379	-2.307809	0.000484
8	3.513266	-0.180176	0.000406
8	-1.169953	2.534683	-0.000364
8	2.096236	-2.513909	-0.000361
6	-1.460247	-0.290407	0.000094
6	-2.288970	0.109224	1.254532
1	-2.514810	1.173953	1.166006
1	-3.240305	-0.440392	1.205948
6	-2.289068	0.108881	-1.254388
1	-3.240399	-0.440725	-1.205586
1	-2.514904	1.173633	-1.166125
6	-1.600240	-0.169363	2.582459
1	-2.250341	0.108232	3.419258
1	-1.338653	-1.226867	2.693219
1	-0.676387	0.409261	2.669005
6	-1.600442	-0.170057	-2.582294
1	-1.338811	-1.227579	-2.692765
1	-2.250633	0.107258	-3.419116
1	-0.676628	0.408591	-2.669096

1.3 Hydroquinone dianion

1.3.1 BHandHLYP/6-31+G(d,p) level

Q1

6	-0.000002	0.698092	1.176328
6	-0.000002	-0.698092	1.176328
6	-0.000051	-1.490311	0.000000
6	-0.000002	-0.698092	-1.176328
6	-0.000002	0.698092	-1.176328
6	-0.000072	1.490311	0.000000
1	0.000044	1.226877	2.123289
1	0.000042	-1.226877	2.123289
1	0.000042	-1.226877	-2.123289
1	0.000044	1.226877	-2.123289
8	0.000096	2.789273	0.000000
8	-0.000020	-2.789273	0.000000

Q2

6	2.630036	-0.706729	0.000006
6	1.436590	-1.382594	-0.000005
6	0.193235	-0.718722	-0.000011
6	0.193235	0.718722	-0.000006
6	1.436591	1.382592	0.000006
6	2.630037	0.706727	0.000012
1	3.565618	-1.249801	0.000010

1	1.398318	-2.460079	-0.000006
6	-1.026502	-1.498269	-0.000014
6	-1.026501	1.498269	-0.000022
1	1.398321	2.460078	0.000005
1	3.565619	1.249798	0.000020
6	-2.180508	0.704490	0.000003
6	-2.180509	-0.704487	-0.000002
1	-3.129717	1.226845	0.000021
1	-3.129719	-1.226842	0.000011
8	-1.018915	2.787779	0.000079
8	-1.018918	-2.787778	-0.000062

Q3

6	-0.595389	2.526747	-0.000010
6	0.597717	1.731878	-0.000011
6	0.385758	0.347588	0.000007
6	-0.952662	-0.239010	0.000013
6	-2.132575	0.600709	0.000011
6	-1.851861	2.016407	-0.000002
1	-0.439395	3.598313	-0.000020
6	-1.114610	-1.669447	0.000020
1	-2.717446	2.665037	-0.000005
6	0.128130	-2.489956	-0.000001
6	1.348212	-1.984942	-0.000006
1	-0.046154	-3.556529	-0.000009
1	2.212045	-2.642502	-0.000016
8	-3.327918	0.214010	0.000001
8	1.754994	2.326474	-0.000016
8	-2.164467	-2.318887	-0.000003
6	1.644352	-0.521654	0.000002
6	2.497180	-0.230862	-1.254172
1	2.728328	0.828072	-1.275197
1	3.422237	-0.815919	-1.237907
1	1.940154	-0.500386	-2.148297
6	2.497185	-0.230881	1.254178
1	3.422242	-0.815938	1.237900
1	2.728331	0.828052	1.275221
1	1.940163	-0.500421	2.148301

Q4

6	1.066956	2.501414	-0.000086
6	-0.196094	1.819800	-0.000073
6	-0.115588	0.422453	0.000012
6	1.163137	-0.285987	0.000052
6	2.416275	0.440005	-0.000014
6	2.269353	1.876946	-0.000044
1	1.010129	3.582644	-0.000139
6	1.194181	-1.725558	0.000157
1	3.191994	2.441280	-0.000055
6	-0.117407	-2.429127	0.000008
6	-1.285251	-1.813634	-0.000018
1	-0.042215	-3.507291	-0.000043
1	-2.205358	-2.390170	-0.000078
8	3.569845	-0.054467	0.000104
8	-1.289631	2.523367	-0.000195
8	2.181501	-2.466742	-0.000049
6	-1.449835	-0.328857	0.000026
6	-2.299416	0.045212	1.245497

1	-2.506525	1.106426	1.164564
1	-3.248216	-0.500108	1.190155
6	-2.299437	0.045277	-1.245408
1	-3.248225	-0.500065	-1.190094
1	-2.506570	1.106481	-1.164396
6	-1.625860	-0.242375	2.579326
1	-2.260617	0.074940	3.408120
1	-1.407510	-1.300959	2.707337
1	-0.686603	0.297393	2.642617
6	-1.625883	-0.242203	-2.579263
1	-1.407520	-1.300776	-2.707352
1	-2.260645	0.075166	-3.408033
1	-0.686631	0.297579	-2.642518

1.3.2 M06-2x/6-31+G(d,p) level

Q1

6	0.000001	-0.701093	1.182645
6	0.000001	0.701093	1.182645
6	-0.000160	1.499330	0.000000
6	0.000001	0.701093	-1.182645
6	0.000001	-0.701093	-1.182645
6	0.000239	-1.499330	0.000000
1	-0.000055	-1.237991	2.133282
1	-0.000002	1.237990	2.133282
1	-0.000002	1.237990	-2.133282
1	-0.000055	-1.237991	-2.133282
8	-0.001345	-2.803713	0.000000
8	0.001298	2.803714	0.000000

Q2

6	2.641988	-0.710075	0.000003
6	1.441822	-1.389002	-0.000007
6	0.194144	-0.721626	-0.000009
6	0.194145	0.721626	-0.000002
6	1.441824	1.389000	0.000008
6	2.641989	0.710072	0.000010
1	3.583291	-1.256611	0.000004
1	1.399579	-2.474667	-0.000010
6	-1.030791	-1.508607	-0.000015
6	-1.030789	1.508608	-0.000011
1	1.399583	2.474666	0.000011
1	3.583292	1.256606	0.000018
6	-2.191096	0.707474	0.000004
6	-2.191097	-0.707470	-0.000004
1	-3.144620	1.236547	0.000017
1	-3.144622	-1.236542	0.000003
8	-1.021831	2.802905	0.000065
8	-1.021836	-2.802904	-0.000053

Q3

6	0.578380	2.542368	0.000007
6	-0.618116	1.737188	-0.000002
6	-0.390781	0.345563	-0.000003
6	0.953699	-0.232208	-0.000008
6	2.137160	0.614719	-0.000013
6	1.845607	2.037054	0.000009
1	0.411437	3.619960	0.000012

6	1.131155	-1.667137	-0.000010
1	2.714462	2.693681	0.000018
6	-0.113648	-2.503256	-0.000002
6	-1.343437	-2.001121	-0.000005
1	0.070209	-3.575859	0.000003
1	-2.214487	-2.662846	-0.000003
8	3.338723	0.226860	0.000026
8	-1.784702	2.324832	-0.000049
8	2.193391	-2.308571	0.000025
6	-1.644859	-0.533317	0.000000
6	-2.499369	-0.247729	1.258221
1	-2.742271	0.816782	1.274461
1	-3.420945	-0.849277	1.244104
1	-1.926445	-0.510708	2.152196
6	-2.499381	-0.247716	-1.258210
1	-3.420960	-0.849256	-1.244086
1	-2.742275	0.816798	-1.274442
1	-1.926468	-0.510693	-2.152193

Q4

6	-1.030661	2.521510	-0.000146
6	0.233234	1.823747	-0.000110
6	0.130856	0.418396	0.000026
6	-1.156897	-0.275322	0.000110
6	-2.411303	0.463486	0.000052
6	-2.247558	1.906928	-0.000122
1	-0.958740	3.609431	-0.000210
6	-1.209021	-1.721237	0.000279
1	-3.171163	2.483691	-0.000188
6	0.103156	-2.445420	0.000073
6	1.283285	-1.835808	0.000022
1	0.015134	-3.530194	0.000014
1	2.208699	-2.419677	-0.000065
8	-3.572808	-0.028343	-0.000180
8	1.341078	2.514881	0.000217
8	-2.212644	-2.450844	-0.000141
6	1.458246	-0.346828	0.000016
6	2.307116	0.016706	-1.252032
1	2.548353	1.078481	-1.164125
1	3.241344	-0.566840	-1.217076
6	2.307182	0.016742	1.252010
1	3.241458	-0.566721	1.216954
1	2.548324	1.078541	1.164121
6	1.579147	-0.241522	-2.569145
1	2.202655	0.038606	-3.426192
1	1.298066	-1.294555	-2.674773
1	0.661315	0.350659	-2.595558
6	1.579351	-0.241603	2.569177
1	1.298350	-1.294660	2.674773
1	2.202920	0.038521	3.426181
1	0.661482	0.350513	2.595707

1.3.3 PBE0/6-31+G(d,p) level

Q1

6	0.000014	-0.700862	1.181563
6	0.000014	0.700862	1.181563
6	0.000162	1.501486	0.000000

6	0.000014	0.700862	-1.181563
6	0.000014	-0.700862	-1.181563
6	0.000429	-1.501487	0.000000
1	-0.000214	-1.236199	2.135871
1	-0.000181	1.236199	2.135871
1	-0.000181	1.236199	-2.135871
1	-0.000214	-1.236199	-2.135871
8	-0.000887	-2.804387	0.000000
8	0.000501	2.804387	0.000000

Q2

6	2.643993	0.709016	-0.000011
6	1.441557	1.386961	-0.000083
6	0.195586	0.723718	-0.000043
6	0.195586	-0.723718	0.000079
6	1.441557	-1.386961	0.000142
6	2.643993	-0.709015	0.000092
1	3.586505	1.258239	-0.000051
1	1.397507	2.474619	-0.000229
6	-1.028061	1.508820	-0.000272
6	-1.028061	-1.508821	0.000289
1	1.397508	-2.474619	0.000287
1	3.586506	-1.258238	0.000150
6	-2.190100	-0.705016	0.000018
6	-2.190100	0.705015	-0.000112
1	-3.146413	-1.233978	-0.000001
1	-3.146413	1.233977	-0.000182
8	-1.026931	-2.801602	-0.000673
8	-1.026931	2.801602	0.000602

Q3

6	-0.563568	2.542710	-0.000113
6	0.623856	1.733921	-0.000270
6	0.390011	0.337136	-0.000006
6	-0.952609	-0.232322	0.000086
6	-2.133684	0.627987	-0.000144
6	-1.834938	2.041727	0.000031
1	-0.392678	3.621568	-0.000127
6	-1.133731	-1.666069	0.000370
1	-2.701398	2.704770	0.000170
6	0.100886	-2.498762	0.000003
6	1.336089	-2.004562	-0.000061
1	-0.087406	-3.572475	-0.000106
1	2.203865	-2.672949	-0.000177
8	-3.338258	0.244465	0.000549
8	1.794113	2.309091	-0.000699
8	-2.204084	-2.305009	-0.000106
6	1.641075	-0.542886	0.000060
6	2.496027	-0.251499	-1.254818
1	2.724284	0.818990	-1.265633
1	3.428511	-0.839700	-1.238378
1	1.933354	-0.520584	-2.155065
6	2.495876	-0.251703	1.255090
1	3.428387	-0.839864	1.238637
1	2.724066	0.818797	1.266144
1	1.933113	-0.520998	2.155218

Q4

6	-1.044422	2.519395	0.000056
6	0.217534	1.829277	0.000067
6	0.122565	0.416223	0.000006
6	-1.158729	-0.280892	-0.000079
6	-2.418007	0.459936	-0.000094
6	-2.259567	1.897141	0.000013
1	-0.978569	3.609524	0.000092
6	-1.201912	-1.726257	-0.000169
1	-3.187037	2.471474	0.000026
6	0.105587	-2.436646	-0.000076
6	1.286827	-1.824538	-0.000014
1	0.022327	-3.523626	-0.000080
1	2.214936	-2.406749	0.000026
8	-3.578808	-0.037607	-0.000007
8	1.322289	2.519881	-0.000075
8	-2.207543	-2.462943	-0.000030
6	1.453254	-0.340261	0.000038
6	2.300417	0.036825	-1.248071
1	2.498749	1.110300	-1.159495
1	3.259680	-0.507341	-1.192892
6	2.300305	0.036723	1.248252
1	3.259547	-0.507488	1.193148
1	2.498693	1.110194	1.159748
6	1.611401	-0.256366	-2.572868
1	2.228856	0.070731	-3.420530
1	1.397559	-1.325137	-2.696191
1	0.656983	0.276959	-2.613638
6	1.611131	-0.256497	2.572960
1	1.397192	-1.325258	2.696197
1	2.228526	0.070501	3.420704
1	0.656750	0.276901	2.613666
